# Ginseng Genome Database: an open-access platform for genomics of *Panax ginseng*

**DOI:** 10.1186/s12870-018-1282-9

**Published:** 2018-04-12

**Authors:** Murukarthick Jayakodi, Beom-Soon Choi, Sang-Choon Lee, Nam-Hoon Kim, Jee Young Park, Woojong Jang, Meiyappan Lakshmanan, Shobhana V. G. Mohan, Dong-Yup Lee, Tae-Jin Yang

**Affiliations:** 1 Department of Plant Science, Plant Genomics and Breeding Institute, Research Institute for Agriculture and Life Sciences, College of Agriculture and Life Sciences, Seoul National University, Seoul, 151-921 Republic of Korea; 2 Phyzen Genome Institute, Seongnam-si, Gyeonggi-do 13558 Republic of Korea; 3 Bioprocessing Technology Institute; Agency for Science, Technology and Research (A*STAR), 20 Biopolis Way, #06-01, Centros, Singapore, 138668 Singapore; 4 Centre for Plant Molecular Biology and Biotechnology, Tamil Nadu Argricultural University, Coimbatore - 03, India; 5 School of Chemical Engineering, Sungkyunkwan University, 2066 Seobu-ro, Jangan-gu, Suwon, Gyeonggi-do 16419 Republic of Korea; 6 Crop Biotechnology Institute, Green Bio Science and Technology, Seoul National University, Pyeongchang, 232-916 Republic of Korea

**Keywords:** *Panax ginseng*, Genome database, Ginseng annotation, Ginseng genome browser

## Abstract

**Background:**

The ginseng (*Panax ginseng* C.A. Meyer) is a perennial herbaceous plant that has been used in traditional oriental medicine for thousands of years. Ginsenosides, which have significant pharmacological effects on human health, are the foremost bioactive constituents in this plant. Having realized the importance of this plant to humans, an integrated omics resource becomes indispensable to facilitate genomic research, molecular breeding and pharmacological study of this herb.

**Description:**

The first draft genome sequences of *P. ginseng* cultivar “Chunpoong” were reported recently. Here, using the draft genome, transcriptome, and functional annotation datasets of *P. ginseng*, we have constructed the Ginseng Genome Database http://ginsengdb.snu.ac.kr/, the first open-access platform to provide comprehensive genomic resources of *P. ginseng*. The current version of this database provides the most up-to-date draft genome sequence (of approximately 3000 Mbp of scaffold sequences) along with the structural and functional annotations for 59,352 genes and digital expression of genes based on transcriptome data from different tissues, growth stages and treatments. In addition, tools for visualization and the genomic data from various analyses are provided. All data in the database were manually curated and integrated within a user-friendly query page.

**Conclusion:**

This database provides valuable resources for a range of research fields related to *P. ginseng* and other species belonging to the Apiales order as well as for plant research communities in general. Ginseng genome database can be accessed at http://ginsengdb.snu.ac.kr/.

## Background

Ginseng (*Panax ginseng* C.A. Meyer) is a perennial herb of the *Panax* genus in Araliaceae family and has widely been used as a traditional medicine in Eastern Asia and North America. The principle bioactive components in ginseng are ginsenosides (collectively a group of triterpene saponins), which are biosynthesized through the isoprenoid pathway [1]. Ginseng has various therapeutic effects on humans including for treatment of cancer, diabetes, cardiovascular and stress [2–6]. *P. ginseng* is known to be tetraploid (2n = 4× = 48), with an estimated genome size of approximately 3.6 Gbp [7, 8]. Its large, highly repetitive genome, which has experienced whole-genome duplication, has impeded the progress of whole-genome sequencing of *P. ginseng* [7]. In addition, the long generation time (4 years) and difficulty of maintenance in ginseng cultivation fields have limited the genetic study of *P. ginseng*. Nevertheless, with the advent of new sequencing technologies, expressed sequence tags (ESTs) and RNA-Seq data have been generated from various tissues and growth stages of *P. ginseng* [9–12], based on which a number of genes involved in ginsenoside biosynthesis pathway have been characterized [10, 11]. Recently, the complete chloroplast genome sequences of *P. ginseng* cultivars and related species were characterized [13, 14]. Furthermore, inter- and intra-species chloroplast genome diversity were also identified for authentication of ginseng cultivars and species [13–17].

At the outset of this project, a total of 17,773 ESTs from NCBI db-EST (as of January, 2017) and a database for adventitious root [9] were publicly available for ginseng. However, these data were insufficient to facilitate the functional and comparative genomics and molecular breeding of ginseng. There was no comprehensive database publicly available for ginseng despite its importance as a medicinal crop with high pharmacological value. Given the fact that ginseng shows numerous effects on human health, a genomic and transcriptomic database is vital for ginseng research communities and other close relatives in the Apiales order. It is also anticipated that an integrated database of genetic, genomic, and metabolomic resources of ginseng would serve as a valuable resource for translational genomics. Recently, we generated extensive genomic and transcriptomic data for *P. ginseng* cultivar “Chunpoong” [18].

In this study, we built a dynamic database that integrates a draft genome sequence, transcriptome profiles, and annotation datasets of ginseng. This Ginseng Genome Database is now publicly available (http://ginsengdb.snu.ac.kr/) for the use of scientific community around the globe for exploring the vast possibilities.

This user-friendly database will serve as a hub for mining gene sequences and their digital expression data of samples from various tissues, developmental stages, and treatments. Our database interface will facilitate the easy retrieval of gene families and associated functional annotations using InterPro, KEGG, BLAST and Gene Ontology (GO) databases. To expedite metabolomics in ginseng, we have made a separate section that categorizes the genes associated with various metabolic pathways including the ginsenoside biosynthesis pathway. In addition, we have included robust tools such as BLAST and genome browser (JBrowse) [19] for survey and visualization of ginseng genomic features. This database will be updated regularly with new genome sequences and information on annotation and will provide reference genomic information for research in *P. ginseng* as well as related species.

## Construction and content

### Whole-genome sequencing and assembly and gene models

The genome sequence data of *P. ginseng* were generated from an elite cultivar ‘Chunpoong’ using Illumina HiSeq platforms. A total of 746 Gb paired-end and 365 Gb mate-paired raw data were produced and assembled, yielding the draft genome sequence of about ~ 3.0 Gb in size. The repeat sequences were identified and masked using RepeatModeler [20] and RepeatMasker [21]. An automatic gene prediction was performed using evidence modeler (EVM) [22] with ab initio predictions (BRAKER 1 [23]), protein evidence, ESTs and RNA-Seq evidence [24]. After the removal of the transposon sequences, a total 59,352 putative protein coding genes were predicted. These genes were functionally annotated using InterPro [25], Blast2Go [26], KEGG [27] and BLASTP searches with known protein databases.

### Transcriptome data

The transcriptome data were generated from various tissues and abiotic stress-treated samples of ginseng using Illumina HiSeq and PacBio platforms (http://ginsengdb.snu.ac.kr/transcriptome.php). Raw RNA-Seq reads of about 120 Gb were pre-processed in four steps to obtain high quality RNA reads. Initially, the bacterial contaminant reads were removed by read mapping against the available bacterial genomes using BWA [28]. After pre-processing, the duplicated reads were filtered out using FastUniq [29]. The third step is the removal of the ribosomal RNA (rRNA) reads using SortMeRNA [30]. Finally, the low-quality reads were removed using NGS QC Toolkit [31]. The high-quality RNA-Seq reads were used for de novo assembly by Trinity [32] and reference-guided assembly by HISAT & stringtie [33] and then for gene prediction on the draft genome sequence. In addition, high quality PacBio sequences were used to refine the predicted gene models.

### Gene families and metabolic pathways

Genes were grouped based on protein domain (Pfam) and InterPro domain. Metabolic pathways were predicted with the KAAS server [27] using the reference information on gene annotation of *Arabidopsis thaliana*, *Citrus sinensis*, *Glycine max*, *Vitis vinifera* and *Solanum lycopersicum*. This information can be accessed at http://ginsengdb.snu.ac.kr/gene_family.php and http://ginsengdb.snu.ac.kr/metabolic_pathway.php.

### Genome-scale metabolic network

Based on gene annotations, a compartmentalized genome-scale metabolic network was reconstructed providing the global overview of all metabolites, enzymes, reactions and pathways in ginseng. This network accounts for a total of 4946 genes, mapped to 2194 enzyme-catalyzed and protein-mediated transport reactions involving 2003 unique metabolites across six intracellular compartments. The global overview of ginseng genome-scale metabolic network can be accessed at http://ginsengdb.snu.ac.kr/network/index.html. This network can also be downloaded as a systems biology markup language (SBML) file.

### Transcription factors

Transcription factors (TFs) are the key regulators for development and stimulus responses. TFs were identified based on the criteria of PlnTFDB [34] using iTAK (http://bioinfo.bti.cornell.edu/cgi-bin/itak/index.cgi). A total of 4439 TF and transcription regulator genes were identified and classified into 94 TF families (http://ginsengdb.snu.ac.kr/tf_class.php).

### Genes in the ginsenoside biosynthesis pathway

Ginsenosides are biosynthesized through the mevalonate (MVA) and 2-C-methyl-D-erythritol-4-phosphate (MEP) pathways [10, 35]. The number of genes that are involved in the biosynthesis of ginsenoside was identified based on KEGG as well as BLASTP annotations. UDP glycosyltransferase (UGT) genes, which are responsible for production of various types of ginsenosides in the final step of this pathway, were also identified based on InterPro ProSitePatterns (PS00375) and BLAST homology searches as well. The putative pathway and the related genes can be accessed at http://ginsengdb.snu.ac.kr/pathway.php.

### Digital gene expression profiles

Digital gene expression profiles were determined using all of the RNA-Seq data. The FPKM values for all genes in each sample were calculated using RSEM [36]. Further, the expression data were normalized using Trimmed Mean of M values (TMM) to resolve the differences in the sequencing depth. The digital expression profiles can be accessed at http://ginsengdb.snu.ac.kr/gene_exp.php.

## Utility and discussion

### Database implementation

**Fig. 1:**
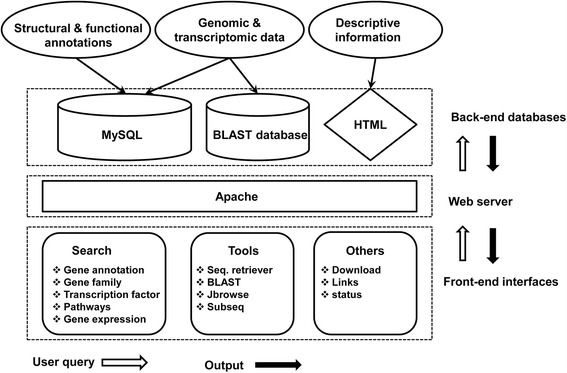
Overview of the architecture of the Ginseng Genome Database

Ginseng Genome Database was established in the Linux (CentOS 6.6) operating system with an Apache HTTP server. PHP, HTML, JavaScript and Python scripts were used to build the user-friendly interface and design web pages. To visualize the genome, we included JBrowse version 1.11.6, which is JavaScript-based genome browser allowing visual analysis of the genome annotation [19]. We also included a BLAST server to perform homology searches with different data sets of ginseng. Moreover, we developed a Python-based tool to retrieve or download specific scaffolds and gene sequences. An overview of the ginseng genome database architecture is shown in Fig. 1.

### Query search

**Fig. 2:**
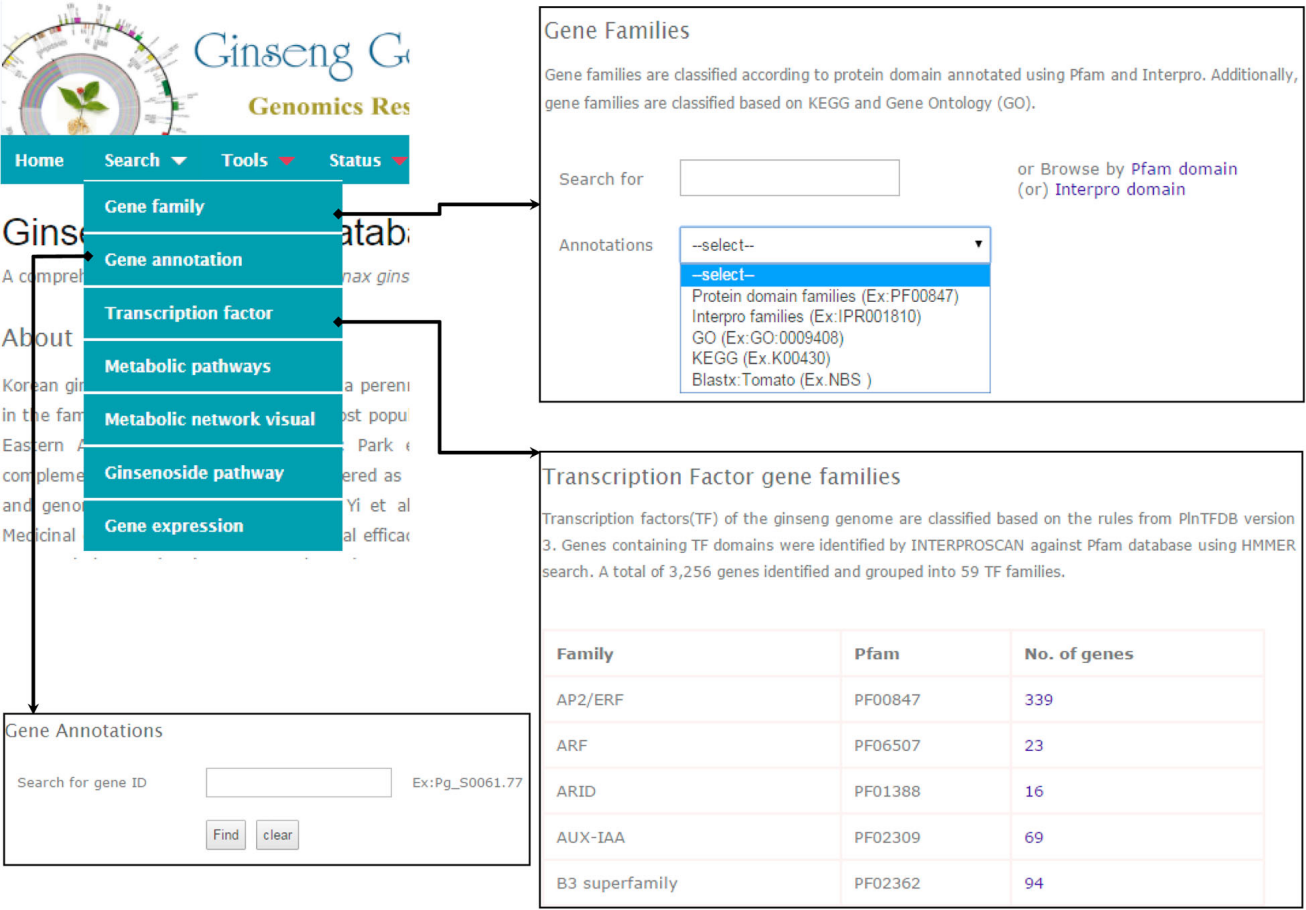
Query interface to retrieve information on gene annotations and transcription factors

**Fig. 3:**
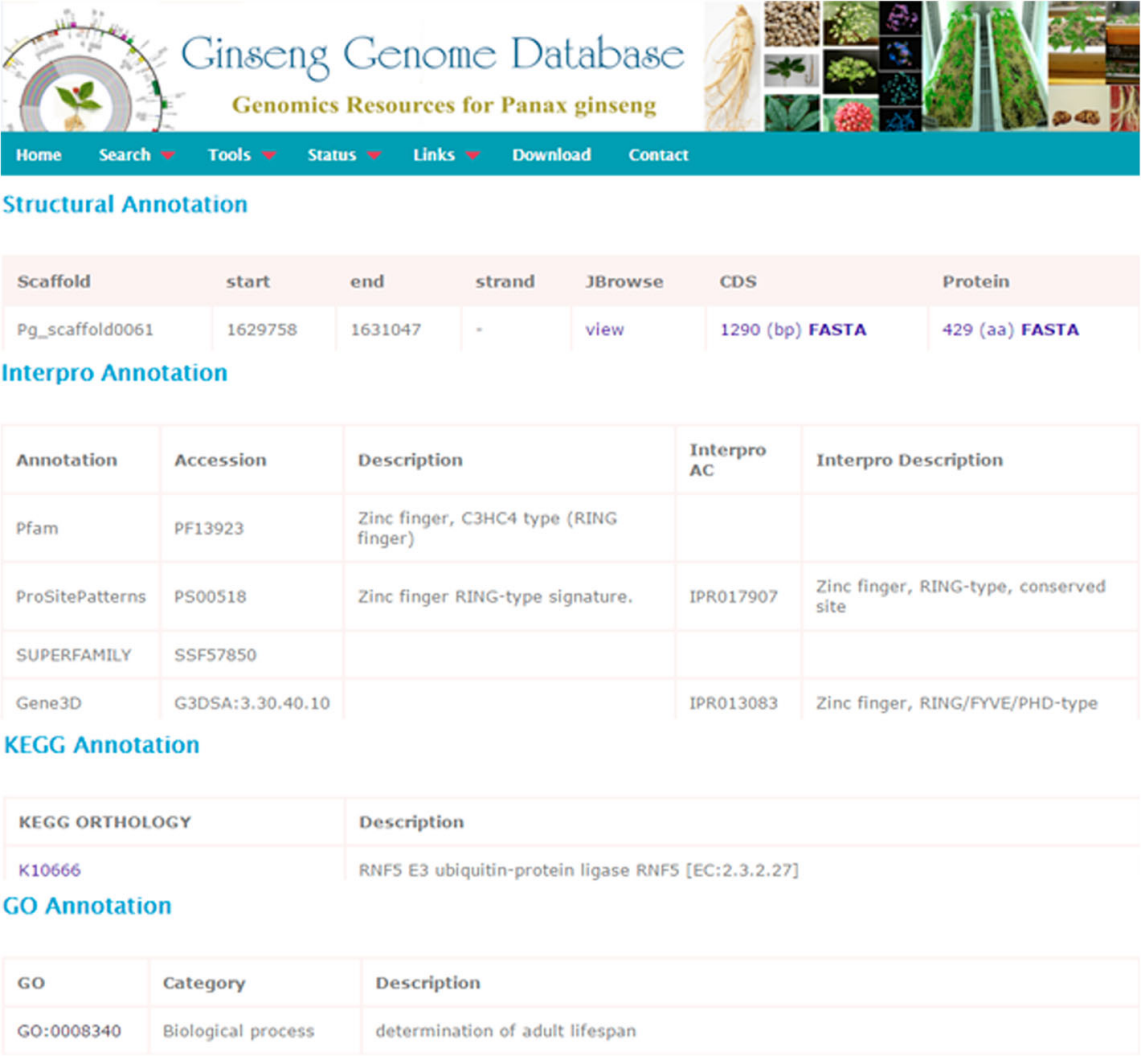
A detailed snapshot of the structural and functional annotations of a queried gene

Ginseng Genome Database provides two major panels, namely, a ‘Search’ panel and a ‘Tool’ panel, both of which comprise of all the information in an easy-to–use mode. Under the ‘Search’ panel, genes or gene families can be searched by gene ID, InterPro domain, Pfam domain, GO and KEGG orthology (KO) identifier (ID) and keywords (Fig. 2). Furthermore, users can browse gene families categorized using ‘InterPro’ and ‘Pfam’ domains. The ‘Gene family’ option provides a sub-menu to retrieve the group of genes related to user-defined functional domains or keywords. Users can download all coding sequences (CDSs) in a specific gene family or user-selected CDSs in FASTA format from the output page. The ‘Gene annotation’ section provides the detailed annotations including both structural and functional annotations of the user-queried genes (Fig. 3). In the output page, users can find the scaffold in which the specified genes, CDS and proteins were annotated and then can visualize those through JBrowse. Further, functional descriptions based on InterPro annotation including Pfam, Prositepatterns, and Superfamily, GO, KEGG and BLAST can also be browsed.

A list of annotated TF families is included in the ‘Transcription factors’ section (Fig. 2). Users can explore the TF genes related to specific TF families and download the corresponding CDSs. Under the ‘Metabolic pathways’ section, the users can simply enter a pathway name or click browse pathways to retrieve the genes involved in a particular pathway. Our database also provides links, so that users can check the ‘enzyme commission (EC) number’ for the corresponding genes and the complete pathway from the KEGG database. The known pathway of biosynthesis of ginsenosides and genes corresponding to each enzyme are listed under the ‘Ginsenoside pathway’ section. In the ‘Gene expression’ section, users can input a specific gene identifier and can choose to compare expressions between ginseng plant tissues or between abiotic stresses. This will return the expression data in bar-chart form using different colors.

### Sequence retriever

We incorporated a ‘sequence retrieving tool’ using Python script. This can be utilized by entering single or batch gene (CDSs and peptides) or scaffold IDs in the input query box. We customized the output options with ‘view’ and ‘download’. Users can view single or multiple sequences in FASTA format on the web page by choosing the ‘view’ option. In case of many sequences, the user may select the ‘download’ option to download the sequences in FASTA format.

### BLAST

This database also offers a homology search tool, ‘BLAST’, which was embedded in the database using the ncbi-wwwblast package (v2.2.26) to provide a graphic interface for the users. A BLAST-able databases of the whole draft genome sequence, coding sequences (CDSs), and protein sequences were made for BLAST searches. Additionally, the transcriptome data from various tissues and abiotic stress treatments of ginseng generated for the whole-genome study, the RNA-Seq assembly that were previously published and the ESTs were provided for BLAST searches. The users can perform BLAST searches by directly pasting the query sequences in the ‘query text box’, by choosing the appropriate search program (BLASTN, BLASTP, BLASTX, TBLASTN or TBLASTX), where BLASTP and TBLASTN are queried only against amino acid sequences. Options to filter low complexity and to set the *E-*value are available under the ‘Other options’ section. The result format can also be customized using the options under ‘Result options’.

### JBrowse

Under the ‘Tools’ panel, ‘JBrowse’ was included to visualize the genomic features of ginseng. All of the assembled scaffolds and the predicted genes were used in constructing the genome browser. The main page of ‘JBrowse’ contains several tracks under different sub-sections. Users can choose the ‘scaffold’ (only 30 scaffolds can be seen in the drop-down menu) or type the name of the scaffold with or without a location in the search box. Users can visualize various genomic features such as ‘gene models’, ‘*ab initio* gene models’ generated for the gene annotation pipeline, ‘assembled transcriptome structure’ and ‘repeats’. In addition, the alignments of RNA-Seq reads to the genome sequence generated directly from Binary Alignment/MAP (BAM) and PacBio contig alignment using GMAP [37] were also incorporated to perk up the structural annotation of the gene. Furthermore, protein sequences of non-coding genes including microRNA (miRNA) and long non-coding RNAs (lncRNA) can be seen along with their gene features. Apart from *Panax ginseng*, we have incorporated the genome-guided transcriptome assembly of other *Panax* species, namely, *P. notoginseng* and *P. quinquefolius* which would aid in comparing the gene structure or find missing genes any other *Panax* species.

### Downloads

All the assembled genomic and transcriptomic sequences are available at http://ginsengdb.snu.ac.kr/data.php. Our database provides HTTP links to download the draft genome sequences (v1) and putative CDSs and protein sequences (v1.1) in FASTA format. The gene and repeat structure annotations are available in Generic File Format (GFF3). The list of data files including de novo and reference-guided transcriptome assembly generated for whole genome study as well as the previously published transcriptome sequences generated from our research are also accessible in FASTA format. Besides, the filtered RNA-Seq data used for genome analysis and the genome-scale metabolic network of ginseng can also be downloaded as a SBML file.

## Conclusions

Ginseng Genome Database, the original, all-inclusive database for ginseng, is built on the most recent information of its draft genome sequence and accurate annotations. It serves as an open-access interface to retrieve genomic information from genome to gene level and to visualize all diverse components of the genome. The Ginseng Genome Database will form a valuable resource enhancing various research fields like functional/comparative genomics, metabolomics, molecular breeding, and evolutionary analysis of ginseng.

## Abbreviations

bp: Base pair
CDS: Coding sequence
EC: Enzyme commission
EST: Expressed sequence tag
EVM: Evidence modeler
FPKM: Fragments per kilobase of exon per million fragments
GFF: General feature format
kb: Kilobase pair
lncRNA: Long noncoding RNA
NGS: Next generation sequencing
TF: Transcription factor
TMM: Trimmed mean of M values
